# Renal cell neoplasias: reversion-inducing cysteine-rich protein with Kazal motifs discriminates tumor subtypes, while extracellular matrix metalloproteinase inducer indicates prognosis

**DOI:** 10.1186/1479-5876-11-258

**Published:** 2013-10-16

**Authors:** Anja Rabien, Carsten Stephan, Ergin Kilic, Wilko Weichert, Glen Kristiansen, Kurt Miller, Klaus Jung, Andreas Erbersdobler

**Affiliations:** 1 Department of Urology, Research Division, Charité - Universitätsmedizin Berlin, Charitéplatz 1, Berlin 10117, Germany; 2 Berlin Institute for Urologic Research, Berlin, Germany; 3 Institute of Pathology, Charité – Universitätsmedizin Berlin, Berlin, Germany; 4 Institute of Pathology, University of Heidelberg and National Center of Tumor Diseases (NCT), Heidelberg, Germany; 5 Institute of Pathology, University Hospital of Bonn, Bonn, Germany; 6 Institute of Pathology, University of Rostock, Rostock, Germany

**Keywords:** RECK, EMMPRIN, Renal cell carcinoma, TMA

## Abstract

**Background:**

Matrix metalloproteinases can promote invasion and metastasis, which are very frequent in renal cell carcinoma even at the time of diagnosis. Knowing the reversion-inducing cysteine-rich protein with Kazal motifs (RECK) as an inhibitor of matrix metalloproteinases and the extracellular matrix metalloproteinase inducer (EMMPRIN) protein as inducer, we aimed to determine their expression, localization and possible antagonistic action in the pathogenesis and progression of renal cell tumors in a retrospective study.

**Methods:**

Tumor and adjacent normal tissues of 395 nephrectomized patients were immunostained for RECK and EMMPRIN on a tissue microarray.

**Results:**

RECK strongly decreased in renal cell carcinoma compared to normal counterparts (Wilcoxon signed rank test, *P* < 0.001), and it discriminated tumor entities showing the highest expression in oncocytomas. EMMPRIN, however, could be significantly correlated to pT stage and Fuhrman grading (Spearman’s correlation coefficient r_s_ = 0.289 and r_s_ = 0.382, respectively). Higher expression of EMMPRIN was associated with decreased overall survival in Kaplan-Meier analysis (*P* < 0.001), and the EMMPRIN level could independently predict survival for cases without metastasis and involvement of lymph nodes. Decreased RECK expression was confirmed by Western blotting in tissue of eight normal/tumor matches of patients after radical nephrectomy, whereas the EMMPRIN pattern appeared to be heterogeneous.

**Conclusions:**

We propose RECK down regulation in renal cell carcinoma to be an early event that facilitates tumor formation and progression. EMMPRIN, however, as a prognostic tumor marker, increases only when aggressiveness is proceeding and could add an additional step to invasive properties of renal cell carcinoma.

## Background

Kidney cancer is not the most common malignancy, but with a five-year survival rate of 70% in the United States [1] the outcome is often poor. In renal cell carcinoma (RCC) which represent the majority of 85-90% of kidney neoplasms [2,3], survival is mostly determined by distal metastases detected in 30% of the patients even at the time of diagnosis [2]. Usually, RCC can be recognized by sonography, but as symptoms are lacking until late stages of the disease, metastasis of RCC is the main problem in therapeutic approaches [2,3]. Due to the resistance of RCC to radio- and chemotherapy, only surgery can be curative if RCC is diagnosed at an early stage [2,3]. Current so-called “targeted therapies” using tyrosine kinase inhibitors, mTOR inhibitors or antiangiogenic antibodies alone or in combinations are able to slightly extend progression-free survival [2,3], but further therapeutic improvements are needed. Decisions for treatment are based on tumor stage and the histological grade [3,4]. For diagnosis of RCC and its subtypes, several immunohistochemical markers have been suggested, but until now, no biomarker is in routine clinical use for prognostic purposes [5].

In search for new, more useful biomarkers to diagnose RCC or to improve prognosis we aimed to determine the (dys-)balance of an endogenous inhibitor of matrix metalloproteinases (MMPs) and an inducer of MMPs, namely reversion-inducing cysteine-rich protein with Kazal motifs (RECK) and extracellular matrix metalloproteinase inducer (EMMPRIN, CD147), which we have shown to be responsible for a dysbalance in urothelial carcinoma of the bladder [6]. Hitherto, nothing is known for RECK in kidney cancer, but several studies exist indicating EMMPRIN as a prognostic marker or overexpressed in RCC [7-10]. Here we assessed RECK and EMMPRIN in Western blot assays and in immunohistochemical staining in 395 matches (394 for EMMPRIN) of renal cell tumor tissue and adjacent normal renal tissue on a tissue microarray (TMA) and related them to each other and to clinicopathological parameters of the patients.

## Methods

### Patients

Tissue of 395 patients which had been radically or partially nephrectomized at the Department of Urology, Charité University Hospital between 1992 and 2004 was used for the TMA study with the permission of the local ethics committee (Ethikausschuss 1 Campus Charité – Mitte, document no. EA1/134/12). Tumor stages were determined according to the latest version of the TNM classification by the International Union against Cancer [11] and tumor grades were reviewed by a single pathologist (A.E.) according to the Fuhrman system. Clinicopathological patient characteristics are listed in Table 1. The median age was 60 years at nephrectomy (range 21–86), 257 (65.1%) patients survived and 138 (34.9%) died within follow-up times from 0 to 194 months (median 112 months). All cases were selected according to availability of the tissue as well as of follow-up data and were not stratified in any way. Additional eight pairs of tissue with the above mentioned conditions were used for Western blots, but from nephrectomies of the years 2009 and 2010.

**Table 1:** RECK and EMMPRIN staining in renal cell tumors related to clinicopathological parameters

**Variable**		**Cases (n / %)**	**RECK expression (number of cases / %)**				*P *value*
			**0**	**1**	**2**	**3**	
Age	≤60 years	197 (49.9)	161 (81.7)	27 (13.7)	8 (4.1)	1 (0.5)	0.580
	>60 years	198 (50.1)	164 (82.8)	22 (11.1)	5 (2.5)	7 (3.5)	
Sex	male	264 (66.8)	212 (80.3)	36 (13.6)	11 (4.2)	5 (1.9)	0.234
	female	131 (33.2)	113 (86.3)	13 (9.9)	2 (1.5)	3 (2.3)	
pT status	pT1-2	265 (67.1)	216 (81.5)	32 (12.1)	10 (3.8)	7 (2.6)	0.250
	pT3-4	130 (32.9)	109 (83.8)	17 (13.1)	3 (2.3)	1 (0.8)	
Fuhrman grade	1-2	348 (88.1)	285 (81.9)	43 (12.4)	12 (3.4)	8 (2.3)	0.338
	3-4	47 (11.9)	40 (85.1)	6 (12.8)	1 (2.1)	0 (0.0)	
Nodal status**	pN0	214 (89.9)	182 (85.0)	25 (11.7)	5 (2.3)	2 (0.9)	0.878
	pN+	24 (10.1)	20 (83.3)	3 (12.5)	1 (4.2)	0 (0.0)	
Metastases***	M0	338 (90.6)	278 (82.2)	41 (12.1)	12 (3.6)	7 (2.1)	0.300
	M1	35 (9.4)	31 (88.6)	3 (8.6)	1 (2.9)	0 (0.0)	
Margin status****	R0	357 (94.4)	291 (81.5)	46 (12.9)	12 (3.4)	8 (2.2)	0.603
	R+	21 (5.6)	18 (85.7)	2 (9.5)	1 (4.8)	0 (0.0)	
Tumor subtype							
Clear cell RCC		322 (81.5)	291 (90.4)	27 (8.4)	3 (0.9)	1 (0.3)	**<0.001**
Papillary RCC		43 (10.9)	23 (53.5)	11 (25.6)	8 (18.6)	1 (2.3)	
Chromophobe RCC		22 (5.6)	10 (45.5)	7 (31.8)	1 (4.5)	4 (18.2)	
Oncocytoma		8 (2.0)	1 (12.5)	4 (50.0)	1 (12.5)	2 (25.0)	
			**EMMPRIN expression (number of cases / %)**				
			**0**	**1**	**2**	**3**	
Age	≤60 years	196 (49.7)	7 (3.6)	57 (29.1)	70 (35.7)	62 (31.6)	0.479
	>60 years	198 (50.3)	8 (4.0)	54 (27.3)	87 (43.9)	49 (24.7)	
Sex	male	263 (66.8)	13 (4.9)	70 (26.6)	102 (38.8)	78 (29.7)	0.797
	Female	131 (33.2)	2 (1.5)	41 (31.3)	55 (42.0)	33 (25.2)	
pT status	pT1-2	265 (67.3)	14 (5.3)	86 (32.5)	112 (42.3)	53 (20.0)	**<0.001**
	pT3-4	129 (32.7)	1 (0.8)	25 (19.4)	45 (34.9)	58 (45.0)	
Fuhrman grade	1-2	347 (88.1)	15 (4.3)	109 (31.4)	141 (40.6)	82 (23.6)	**<0.001**
	3-4	47 (11.9)	0 (0.0)	2 (4.3)	16 (34.0)	29 (61.7)	
Nodal status**	pN0	213 (89.9)	6 (2.8)	63 (29.6)	78 (36.6)	66 (31.0)	**0.003**
	pN+	24 (10.1)	1 (4.2)	1 (4.2)	7 (29.2)	15 (62.5)	
Metastases***	M0	337 (90.6)	15 (4.5)	101 (30.0)	138 (40.9)	83 (24.6)	**<0.001**
	M1	35 (9.4)	0 (0.0)	6 (17.1)	9 (25.7)	20 (57.1)	
Margin status****	R0	356 (94.4)	15 (4.2)	105 (29.5)	145 (40.7)	91 (25.6)	**0.004**
	R+	21 (5.6)	0 (0.0)	3 (14.3)	6 (28.6)	12 (57.1)	
Tumor subtype							
Clear cell RCC		322 (81.7)	9 (2.8)	91 (28.3)	128 (39.8)	94 (29.2)	0.813
Papillary RCC		42 (10.7)	6 (14.3)	14 (33.3)	16 (38.1)	6 (14.3)	
Chromophobe RCC		22 (5.6)	0 (0.0)	5 (22.7)	9 (40.9)	8 (36.4)	
Oncocytoma		8 (2.0)	0 (0.0)	1 (12.5)	4 (50.0)	3 (37.5)	

*Chi-square test using linear-by-linear association, significant *P* values <0.05 are given in bold. **157 cases were pNx or not assessed, pN+ includes all N-positive cases. ***22 cases were Mx or not assessed. ****17 cases were Rx.

### Tissue microarray

Areas of renal cell tumors and adjacent normal tissue were marked on 3 μm HE stained sections by a board certified pathologist (G.K.). Three tumor and two normal tissue cores per case (1.0 mm diameter) were punched out of the tissue blocks according to marked areas and embedded into a new paraffin block as TMA with up to 37 cases per block. Punching was done with a tissue arrayer (Beecher Instruments, Woodland, CA, USA).

### Immunohistochemistry

TMA sections of 2–3 μm were deparaffinized with xylene, gradually hydrated and cooked in 0.01 M citrate buffer for 5 minutes. EnVision + Dual Link System-HRP (DAB+), Cat. No. K4065 (DAKO, Hamburg, Germany) was used for the staining procedure. Endogenous peroxidase activity and non-specific binding were blocked with the Dual Endogenous Enzyme Block reagent (DAKO) for 10 minutes at room temperature. Primary antibody was incubated for 40 minutes at room temperature using RECK rabbit monoclonal antibody [6,12] 1:250 (clone D8C7, Cat. No. 3433, Cell Signaling Technology Inc., Boston, MA, USA) or EMMPRIN rabbit polyclonal antibody [6,13] 1:500 (Cat. No. 34–5600, Invitrogen, Karlsruhe, Germany). Secondary antibodies conjugated to horseradish peroxidase labelled polymer (DAKO) were applied for 40 minutes at room temperature, followed by staining with 3,3′-diaminobenzidine (DAB+) substrate chromogen (DAKO) and counterstaining with hematoxilin. The optimal concentration of primary antibody had been determined in dilution series on test sections of larger tissue areas. Positive and negative controls guaranteed persistent quality of the immunostaining. RECK and EMMPRIN staining were examined within a range of negative (0) over weak (1) and moderate (2) to strong (3) by a pathologist and a scientist who were blinded for patient outcome as an average for spots of the same case and morphology. Equivocal cases were discussed at a double-headed microscope to reach consent.

### Western blots

Western blots were performed as described before [6]. Briefly, protein concentration of tissue lysates was determined using the Pierce Microplate BCA Protein Assay Kit (Pierce Biotechnology, Rockford, IL, USA). Twenty μg of protein each were separated on a 7.5% (RECK) or 10% (EMMPRIN) sodium dodecylsulfate polyacrylamide gel and transferred onto a polyvinylidene difluoride membrane (Millipore Corp., Bedford, MA, USA). Primary antibodies were the same as mentioned above (immunohistochemistry) and used 1:1,000 (RECK) or 1:5,000 (EMMPRIN) for 1 h at room temperature. The secondary antibody was horseradish peroxidase-conjugated goat anti-rabbit immunoglobulin G (DAKO), diluted 1:2,000. Enhanced chemiluminescence marked bands were detected in a Fluor-S MultiImager (Bio-Rad Laboratories, Hercules, CA, USA). After stripping, beta-actin controls were done as follows: primary monoclonal mouse antibody to beta-actin (Sigma-Aldrich, Munich, Germany) 1:50,000 and as secondary antibody horseradish peroxidase-conjugated rabbit anti-mouse immunoglobulin G (DAKO), diluted 1:5,000.

### Statistical analysis

Calculations were performed using SPSS for Windows 19 (SPSS Inc., Chicago, IL, USA). Associations were determined in bivariate correlation according to Spearman and in chi-square tests using linear-by-linear association. Differences between tumor and adjacent normal tissue were analyzed by the Wilcoxon signed rank test for paired data and the power of discrimination between malignant and non-malignant was calculated in logistic regression analysis. Overall survival analysis was done using Kaplan-Meier analysis and the log-rank test. The tests were two-sided, significance was defined as *P* < 0.05.

## Results

### Expression of RECK and EMMPRIN in renal cell carcinoma

**Figure 1:**
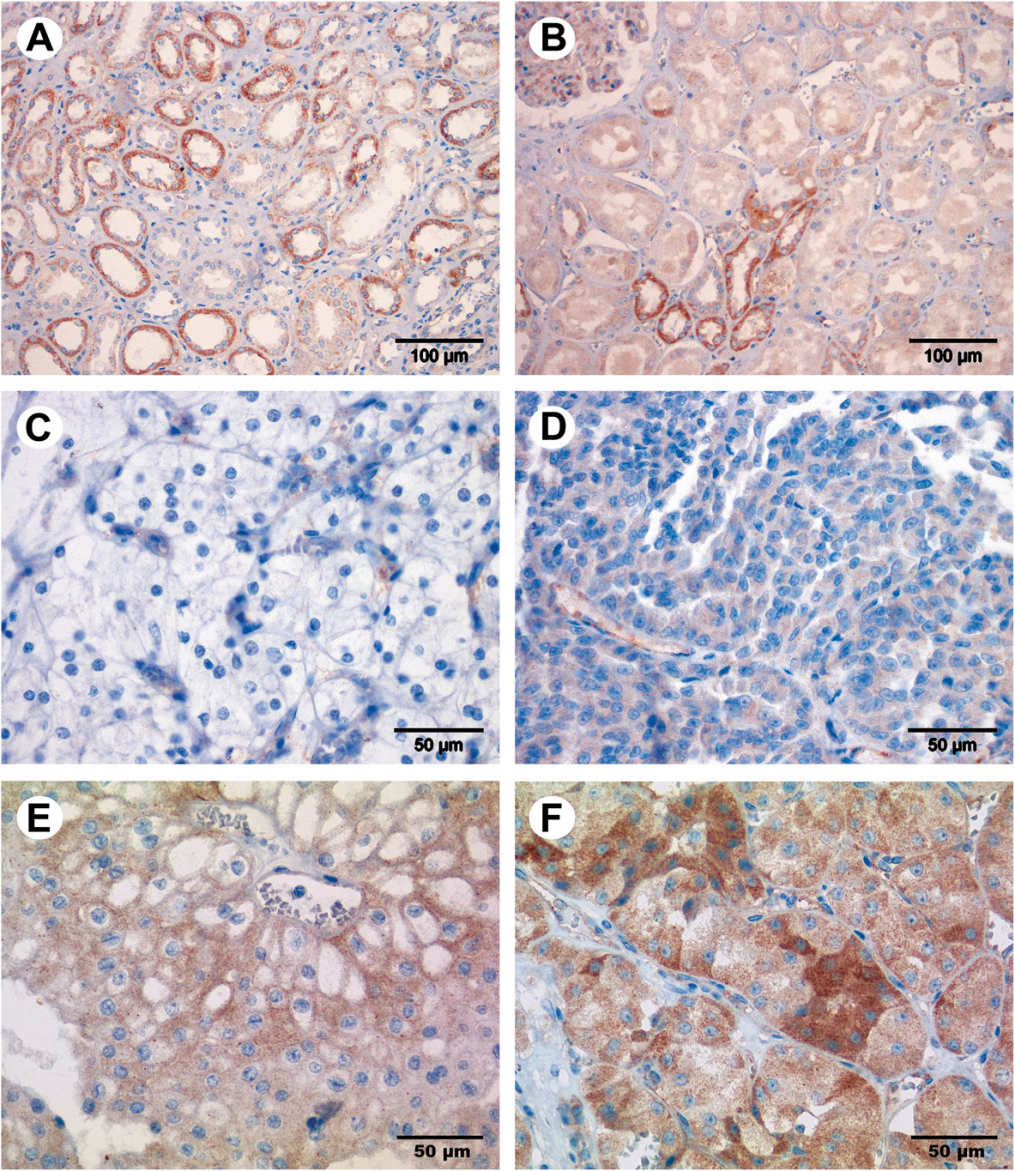
**Immunostaining of RECK in renal cell tissue.** RECK expression was mainly seen in the tubuli and in the capillaries of the glomeruli shown here in the medulla of non-malignant kidney tissue **(A)** and in the non-malignant renal cortex **(B)**. RECK expression increased from clear cell carcinoma **(C)** over papillary **(D)** and chromophobe **(E)** carcinoma to oncocytoma **(F)**. Magnification: 200× **(A**, **B)**, 400× **(C**-**F)**.

**Figure 2:**
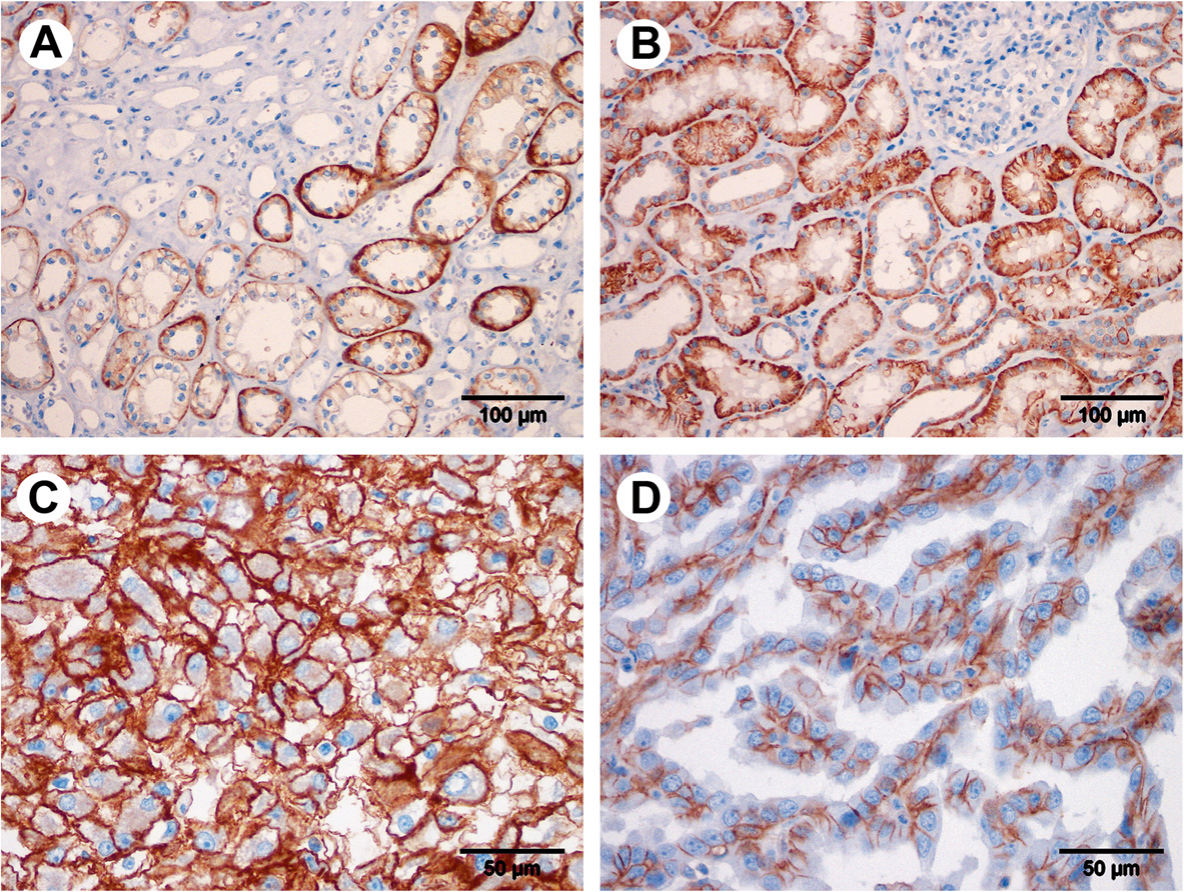
**Immunostaining of EMMPRIN in renal cell tissue.** EMMPRIN expression was detected in the tubuli of non-malignant renal tissue as shown in the medulla **(A)** and in the cortex of the kidney **(B)**. Clear cell **(C)** and papillary **(D)** carcinoma tissues are shown exemplary for tumor staining. Magnification: 200× **(A**, **B)**, 400× **(C**, **D)**.

RECK expression was assessed in 395 cases and EMMPRIN expression in 394 cases of renal neoplasms on the TMA. Clinicopathological data of the patients are given in Table 1 combined with staining intensities of the targets. RECK staining was found in tumor-surrounding normal tissue in the medulla (Figure 1A) as well as in the renal cortex (Figure 1B). Distal tubuli were stained stronger than proximal tubuli, and in the glomeruli only capillaries were stained. RECK expression in normal and tumor cells appeared mainly granularly in the cytoplasm. Interestingly, the average staining intensity increased from clear cell carcinomas over papillary and chromophobe carcinomas to oncocytomas (Figure 1C-F) as proved by linear-by-linear association in a chi-square test (Table 1). This was the only significant association for RECK to clinicopathological parameters. EMMPRIN expression, however, was mainly membranous (Figure 2) and did not discriminate tumor subtypes (Table 1). It was also detected in both, the medulla (Figure 2A) and cortex of the kidney (Figure 2B), but with stronger staining in the proximal tubuli than in the distal ones and absent staining in the glomeruli. Positive and negative controls for immunohistochemical staining are given in an additional figure (Additional file 1).

### Association of EMMPRIN staining with clinicopathological data

EMMPRIN expression in 394 renal neoplasms was bivariately correlated to clinicopathological parameters (listed in Table 1) according to Spearman, Spearman’s rank correlation coefficient r_s_ > 0.200 was considered relevant. EMMPRIN correlated significantly (*P* < 0.001) with pT staging (r_s_ = 0.289) and with Fuhrman grading (r_s_ = 0.382). Associations of the data in chi-square tests confirmed these interrelations (Table 1). Moreover, positive associations were found to lymph node involvement (pN0 versus pN+, *P* = 0.003), metastasis (M0 versus M1, *P* < 0.001) and margin status (R0 versus R+, *P* = 0.004; Table 1).

### Tumor-specificity of RECK

**Figure 3:**
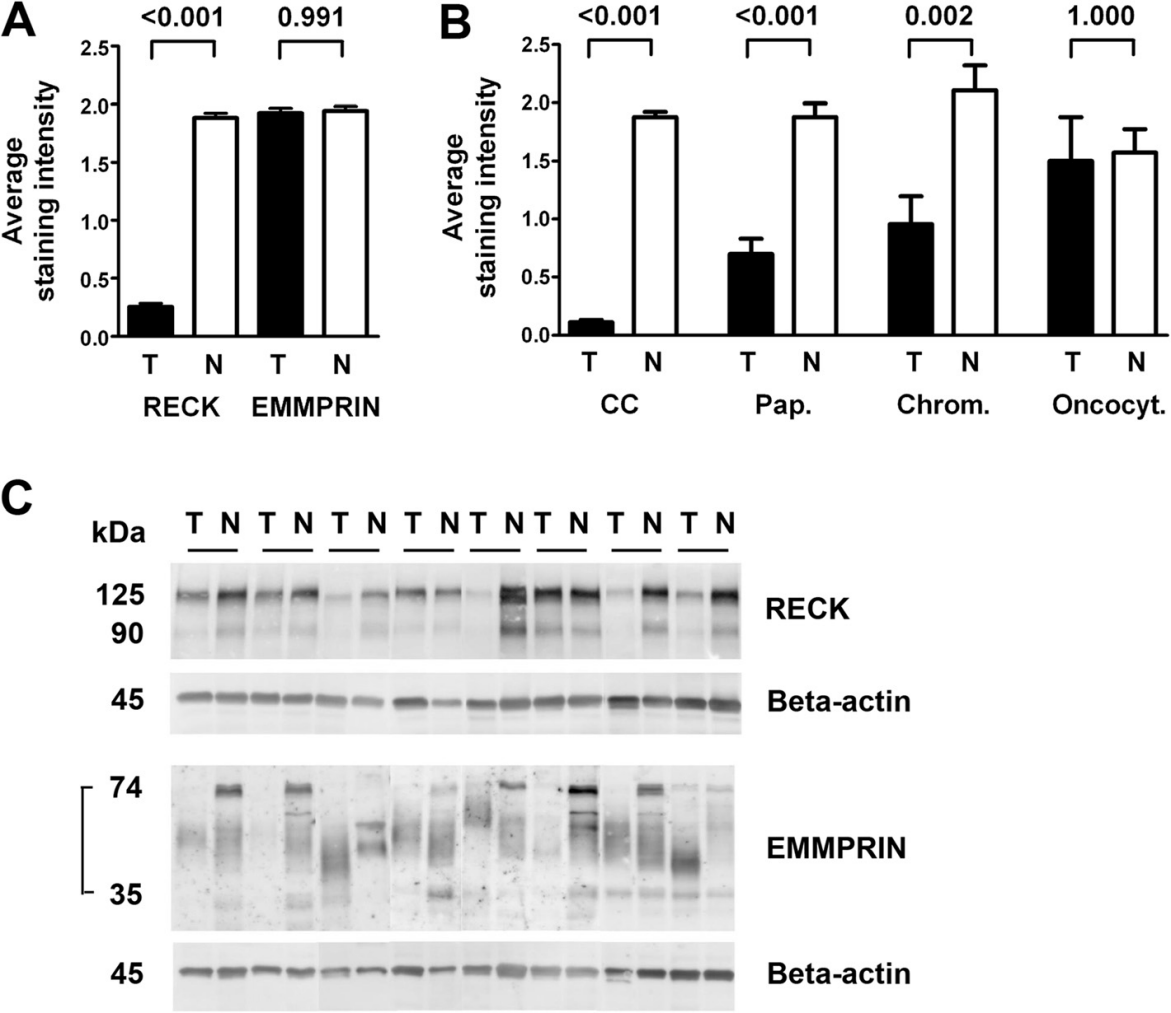
**RECK expression is tumor-specific.** The average staining intensity for RECK decreased to 13% in tumor tissue compared to normal tissue, whereas EMMPRIN staining remained unchanged **(A)**. Mean RECK staining intensities for the tumor subtypes ± standard error of the mean are shown in **(B)**. In comparison to their normal counterparts, RECK values increased gradually from 6% in clear cell carcinomas (CC) over 38% in papillary carcinomas (Pap.) and 45% in chromophobe carcinomas (Chrom.) to 96% in oncocytomas (Oncocyt.). P values are given above the pairs of columns **(A**, **B)**. RECK and EMMPRIN antibodies were checked in Western blots **(C)**. RECK bands at 125 kDa and 90 kDa were detected in protein lysates of 8 pairs of tumor and adjacent normal renal tissue (control: beta-actin). These samples were also used to show the panel of EMMPRIN bands, in our blot migrating between 35 and 74 kDa (control: beta-actin).

Comparison of tumor and adjacent normal tissue pairs revealed a significant decrease of RECK expression in the tumor areas (Wilcoxon matched-pairs signed rank test, *P* < 0.001), whereas expression of EMMPRIN remained unchanged (*P* = 0.991, Figure 3A). A vast majority of 82% of the carcinomas was assessed to be RECK-negative so that further analyses concerning associations and survival analyses failed to become significant. All staining results for RECK and EMMPRIN are shown in Table 2. Logistic regression was performed to further determine the power of RECK to discriminate between tumor and normal renal tissue. With an overall correct classification value of 88.7% RECK discriminated the two groups very well, which was also reflected in the receiver operating curve analysis (curve not shown). The area under the curve (AUC) was calculated as 0.925 with a confidence interval from 0.904 to 0.945.

**Table 2:** RECK and EMMPRIN staining in renal tumors

**RECK staining score**	**Tumor**	**Adjacent normal**	**EMMPRIN staining score**	**Tumor**	**Adjacent normal**
	**Cases (%)**	**Cases (%)**		**Cases (%)**	**Cases (%)**
0	325 (82.3)	17 (4.5)	0	15 (3.8)	6 (1.7)
1	49 (12.4)	92 (24.5)	1	111 (28.2)	93 (25.7)
2	13 (3.3)	185 (49.2)	2	157 (39.8)	179 (49.4)
3	8 (2.0)	82 (21.8)	3	111 (28.2)	84 (23.2)
Total	395 (100)	376 (100)	Total	394 (100)	362 (100)
Mean value	0.3	1.9	Mean value	1.9	1.9
Median	0.0	2.0	Median	2.0	2.0

RECK staining intensity discriminated the tumor subtypes as mentioned above, increasing gradually from clear cell carcinoma to oncocytoma (see also Table 1), but with constantly high levels in the normal tissue (Figure 3B). All carcinoma subtypes, except for oncytoma, showed significant differences in RECK expression between tumor and matched normal tissues (Wilcoxon matched-pairs signed rank test; *P* < 0.001 for clear cell and papillary carcinoma; *P* = 0.002 for chromophobe carcinoma; *P* = 1.000 for oncocytoma). Western blots also presented less amounts of RECK in the tumor tissue (Figure 3C). Eight pairs of renal cell carcinoma and adjacent normal tissue presented the active glycosylated form of RECK migrating at 125 kDa [12] and a lower RECK band of about 90 kDa. EMMPRIN was detected with bands of different molecular weight between about 74 kDa and 35 kDa which represent differentially glycosylated forms of EMMPRIN.

### Survival analysis with RECK and EMMPRIN

**Figure 4:**
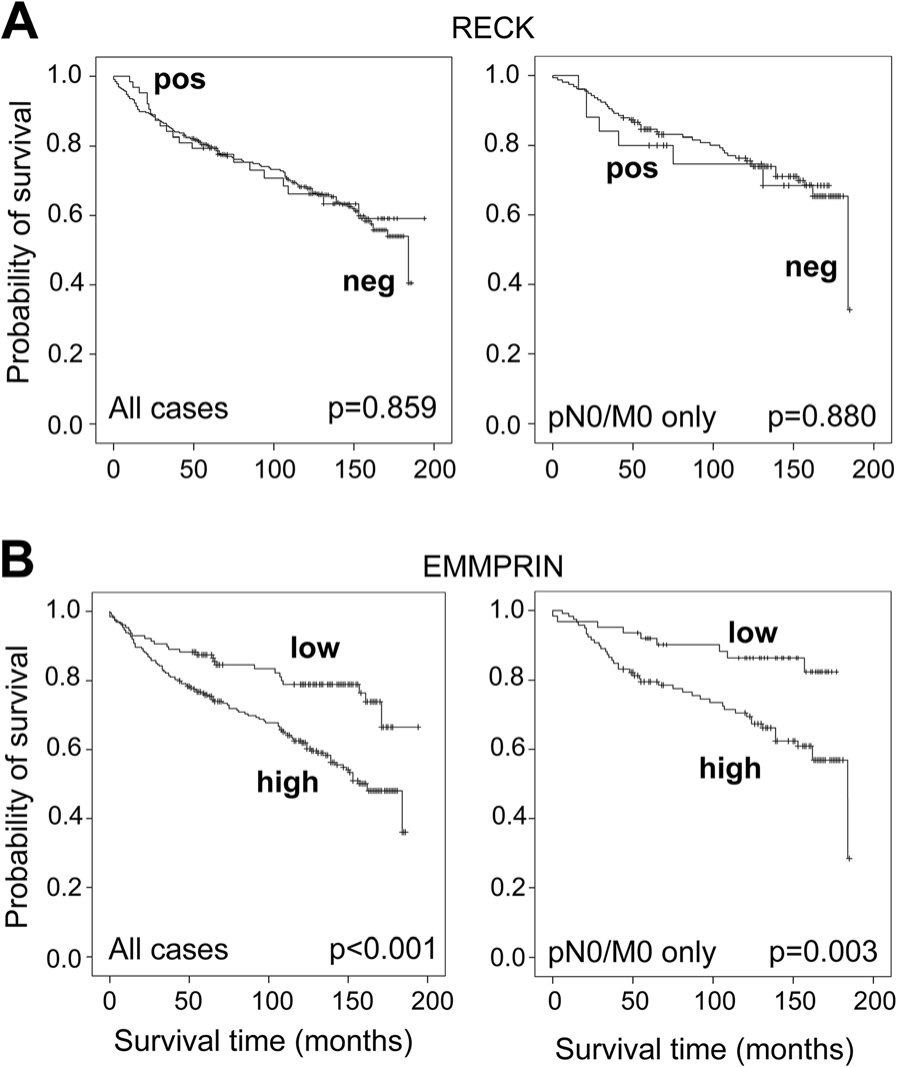
**EMMPRIN is a prognostic marker in renal cell carcinoma.** Kaplan-Meier analyses show RECK-positive (0) and RECK-negative (1–3) staining intensities **(A)** or low EMMPRIN (0/1) and high EMMPRIN (2/3) staining intensities **(B)** connected with overall survival of the patients after nephrectomy. Oncocytomas were excluded. Curves of all 387 cases (386 for EMMPRIN) are shown at the left, curves of the selected 181 pN0/M0 cases only (180 for EMMPRIN) are depicted at the right. High EMMPRIN expression was significantly associated with a decrease in overall survival, even in pN0/M0 cases only, while RECK did not discriminate subgroups. Censored cases are marked (+).

Univariate overall survival analysis according to Kaplan-Meier confirmed the study cohort to be representative, because the important tumor parameters pT stage, Fuhrman grade, nodal status (pN), metastases before surgery (M), and surgical margin status (R) significantly differentiated low risk cases with an advantage in survival from high risk cases with shorter survival times (*P* < 0.001 for all parameters). Oncocytomas (8 cases) had been excluded from survival analyses to avoid a possible bias due to benignity. Data were dichotomized as mentioned above or as negative/positive (pN, M, R). RECK expression in renal carcinoma did not differentiate according to survival time, either in the whole group of 387 cases or in the subgroup of 181 pN0/M0 cases (Figure 4A). EMMPRIN expression, however, clearly discriminated cases with low EMMPRIN expression and an advantage in survival (127 cases, 27 events) from cases with high EMMPRIN expression and shorter survival times (259 cases, 109 events, *P* < 0.001, Figure 4B left). The five-year survival rate decreased accordingly from 87.4% to 75.9%. Using 180 cases of pN0/M0 only, we obtained similar results with 62 cases of low expression (9 events) and 118 cases of high expression (44 events, *P* = 0.003, Figure 4B right) and five-year survival rates of 91.9% and 79.5%, respectively. Kaplan-Meier analyses for non-dichotomized EMMPRIN data show the gradual decrease of survival time with increasing EMMPRIN levels for all cases as well as for the pN0/M0 cases, but they should be considered with reservation due to partially small subgroups (Additional file 2). Even for the pN0/M0 cases multivariate analyses using the Cox proportional hazards regression model emphasized EMMPRIN as an independent predictor of survival (Table 3). Univariate analyses of RECK, EMMPRIN and the clinicopathological parameters sex, age, pT stage, Fuhrman grade and surgical margin status R resulted in the four significant factors age, pT, R and EMMPRIN, which remained significant in the multivariate inclusion model as well as in the backward likelihood calculation, besides R (Table 3). RECK expression again remained irrelevant for survival prognosis as already shown in the Kaplan-Meier analyses. Even considering RECK in adjacent normal renal tissue or in the difference tumor/normal tissue, there was no relevant association. All results were comparable if only the major subgroup of clear cell RCC was selected.

**Table 3:** Cox regression analyses of clinicopathological factors, RECK and EMMPRIN in 181 cases* of pN0/M0 renal carcinoma

**Variable**	**Univariate analyses**		**Multivariate analyses (RECK)**				**Multivariate analyses (EMMPRIN)**			
			**Inclusion**		Backward**		**Inclusion**		Backward**	
	**HR (95% CI)**	***P***	**HR (95% CI)**	***P***	**HR (95% CI)**	***P***	**HR (95% CI)**	***P***	**HR (95% CI)**	***P***
Sex	0.764	0.361	0.766	0.405			0.745	0.356		
Male / female	(0.429-1.362)		(0.409-1.434)				(0.398-1.393)			
Age	1.047	0.006	1.047	0.008	1.048	0.006	1.049	0.006	1.046	0.009
continous	(1.013-1.081)		(1.012-1.083)		(1.013-1.083)		(1.014-1.086)		(1.011-1.082)	
pT stage	2.800	<0.001	2.595	0.001	2.958	<0.001	2.521	0.002	2.581	0.002
pT1-2 / pT3-4	(1.628-4.817)		(1.443-4.669)		(1.690-5.175)		(1.397-4.548)		(1.434-4.643)	
Fuhrman grade	1.569	0.242	1.467	0.365			1.130	0.779		
G1-2 / G3-4	(0.738-3.333)		(0.640-3.363)				(0.481-2.656)			
Surgical margin status***	5.818	0.001	2.342	0.143			2.825	0.078	2.879	0.068
R negative / positive	(2.006-16.879)		(0.750-7.308)				(0.891-8.953)		(0.926-8.950)	
RECK expression	1.063	0.880	1.040	0.930						
negative / positive	(0.479-2.358)		(0.431-2.510)							
EMMPRIN expression*	2.815	0.005					2.538	0.014	2.546	0.012
low (0,1) / high (2/3)	(1.372-5.776)						(1.208-5.333)		(1.229-5.277)	

*EMMPRIN expression in 180 cases: 153 clear cell, 17 papillary (18 for RECK), 10 chromophobe renal cell carcinomas. Oncocytomas were excluded due to benignity. **Reduced model following backward Likelihood elimination (*P* = 0.05 for entry and *P* = 0.10 for removal). ***175 cases, 6 cases were Rx. Abbreviations: *HR* Hazard ratio, *CI* Confidence interval, *PP* value.

## Discussion

To our knowledge, nothing is known about the tumor suppressor RECK in renal cell carcinoma. Due to our findings of RECK/EMMPRIN imbalance in urothelial bladder carcinoma [6], which could promote invasion processes, we decided to look not only at RECK, but also at the EMMPRIN counterpart in renal cell carcinoma. RECK and EMMPRIN were mostly localized as expected. RECK was found with cytoplasmic granular staining as shown for prostate carcinoma [14], urothelial bladder carcinoma [6] and colorectal carcinoma [15], although membranous staining described for the other tumor entities was not prominent. EMMPRIN, however, was mainly localized at the plasma membrane, consistent with our findings for urothelial bladder carcinoma [6] and colorectal carcinoma [16] and consistent with other studies, among them studies on human kidney tissue [9,17] and RCC [7,8,10,18].

We did not find any difference in staining intensities of EMMPRIN between tumor and adjacent normal tissue. This is in contrast to previous studies on RCC which presented low or undetectable levels of EMMPRIN in normal renal epithelia [7,8,10,18], possibly due to the use of antibodies with different epitopes. Nevertheless, our findings are supported by Shimada et al. [17] who detected EMMPRIN in tubular renal cells, but not in glomeruli. Furthermore, our EMMPRIN antibody proved to be specific, as shown in our Western blot experiments and in the literature [13] so that we could not confirm the diagnostic potential for EMMPRIN in kidney cancer research. RECK, however, contains diagnostic potential as we could demonstrate a strong decrease in RCC as compared to adjacent normal tissue. This decrease was more pronounced than in prostate carcinoma [14,19] and fits also well to findings for colorectal cancer [15] and other tumor entities (reviewed in [20,21]).

We found increasing RECK levels in different renal neoplasms, from clear cell carcinoma over papillary to chromophobe carcinoma and oncocytoma, in which RECK expression became similar for tumor and normal tissue. A possible explanation could be different points of origin, because clear cell and papillary carcinoma are supposed to originate from proximal tubules [22] (but perhaps distal tubules, too [4]) and chromophobe carcinoma and oncocytoma from intercalated cells of the collecting duct [4,23]. Higher RECK levels could also contribute to decreasing malignancy up to the benign oncocytoma, but it would be considered as an early marker for malignancy due to the high percentage of RECK-negative tumors in our study.

Several studies on EMMPRIN in RCC indicate a positive correlation with pT stage [7,8,10,18] and Fuhrman grade [7,8,10,18]. Decreased survival rates were shown with higher EMMPRIN levels in Kaplan-Meier analyses [7,8,10,18]. We confirmed these EMMPRIN characteristics for RCC in our work with a larger number of cases (386 versus 52–100) and furthermore, we found EMMPRIN as an independent predictive marker for pN0/M0 cases. Independence in prognosis was shown before for up to 53 patients regarding EMMPRIN alone in all stages of RCC [7,8,10,18] or in combination with vascular endothelial growth factor in advanced RCC [7,8,10,18]. Therefore, our study gives additional information on the clinically interesting group of cases without evident progression which could benefit from a new marker independent from clinicopathological characteristics.

## Conclusions

In summary, we propose RECK as a diagnostic marker for RCC and the subtypes of RCC suggesting that RECK decrease is an early step in tumorigenesis, whereas EMMPRIN as a prognostic marker is a later event associated with increasing aggressiveness.

## Competing interests

The authors declare that they have no competing interests.

## Authors’ contributions

AR and CS designed the study, AR also did writing of the manuscript, analysis and interpretation of the data, and assessed staining intensities. EK captured images of the staining. WW supervised TMA generation and revised the manuscript. GK marked tissue areas for punching. KM revised the manuscript in a clinical point of view. KJ supervised the study, participated in statistical analysis and interpretation of the data and revised the manuscript. AE supervised the study especially for pathological concerns, ranged the tumors in Fuhrman grading, assessed staining intensities, captured staining images and revised the manuscript. All authors read and approved the final manuscript.

## Supplementary Material

Additional file 1 **Quality control for immunohistochemical staining.** Negative and positive controls for RECK and EMMPRIN staining are shown. Click here for file

Additional file 2 **Overall survival time gradually decreases with increasing EMMPRIN expression in renal cell carcinoma.** Kaplan-Meier analyses give additional information on EMMPRIN stepwise discriminating survival groups, even in the pN0/M0 category. Click here for file
