# Supplementary material for: Renal cell neoplasias: reversion-inducing cysteine-rich protein with Kazal motifs discriminates tumor subtypes, while extracellular matrix metalloproteinase inducer indicates prognosis
Source: J Transl Med. 2013 Oct 16;11:258. doi: 10.1186/1479-5876-11-258 (PMC3853196; doi:10.1186/1479-5876-11-258)
Supplement: Additional file 1 — Quality control for immunohistochemical staining. Negative and positive controls for RECK and EMMPRIN staining are shown. [file 1479-5876-11-258-S1.pdf]

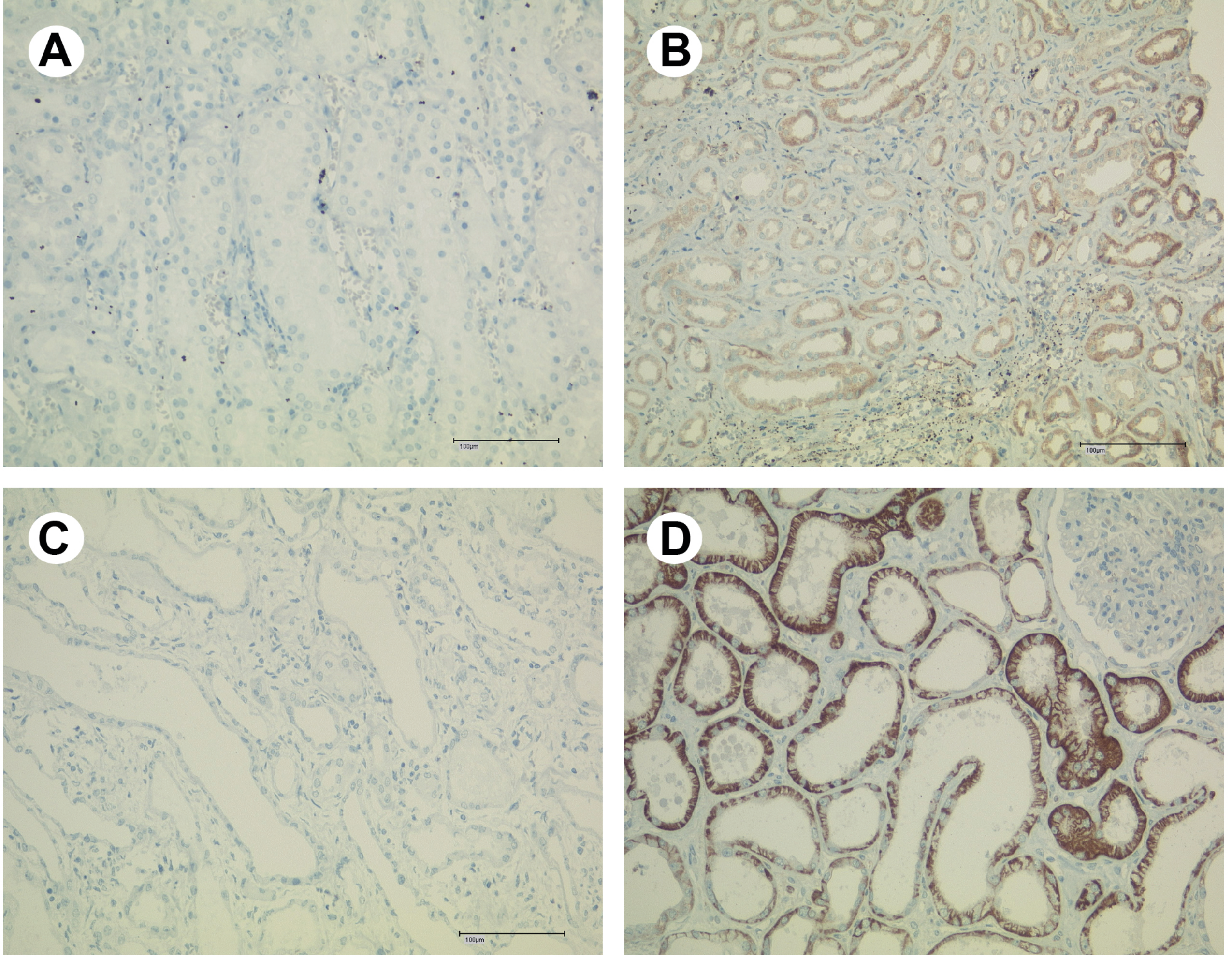

Additional file 1: Quality control for immunohistochemical stainings.
Negative control: Staining of renal tissue without primary antibody is shown for RECK (A) and EMMPRIN (C). Positive control: RECK (B) and EMMPRIN (D) staining are shown for a well known renal tissue sample. Magnification: 200x.
