# Supplementary material for: Renal cell neoplasias: reversion-inducing cysteine-rich protein with Kazal motifs discriminates tumor subtypes, while extracellular matrix metalloproteinase inducer indicates prognosis
Source: J Transl Med. 2013 Oct 16;11:258. doi: 10.1186/1479-5876-11-258 (PMC3853196; doi:10.1186/1479-5876-11-258)
Supplement: Additional file 2 — Overall survival time gradually decreases with increasing EMMPRIN expression in renal cell carcinoma. Kaplan-Meier analyses give additional information on EMMPRIN stepwise discriminating survival groups, even in the pN0/M0 category. [file 1479-5876-11-258-S2.pdf]

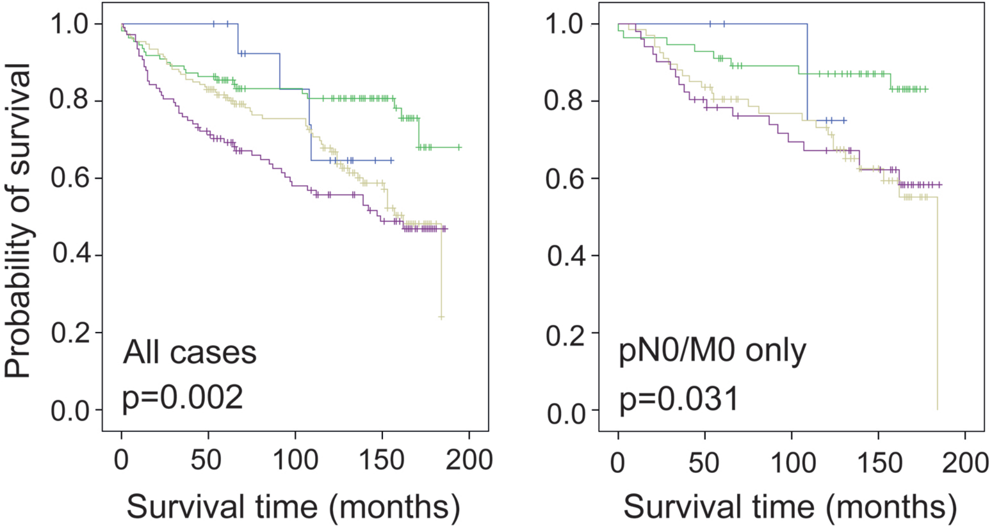

Additional file 2: Overall survival time gradually decreases with increasing EMMPRIN expression in renal cell carcinoma.
Kaplan-Meier analysis shows EMMPRIN staining related to overall survival time after nephrectomy for all 386 cases (left) and for the 180 pN0/M0 cases only (right). Curves are colored in blue for negative staining (0), green for weak (1), beige for moderate (2) and purple for strong (3) staining. Oncocytomas were excluded due to benignity. Censored cases are marked (+).
